# 2-(Arylamino)-6-(trifluoromethyl)nicotinic Acid Derivatives: New HIV-1 RT Dual Inhibitors Active on Viral Replication

**DOI:** 10.3390/molecules25061338

**Published:** 2020-03-15

**Authors:** Angela Corona, Valentina Onnis, Claudia Del Vecchio, Francesca Esposito, Yung-Chi Cheng, Enzo Tramontano

**Affiliations:** 1 Department of Life and Environmental Sciences, University of Cagliari, Cittadella Universitaria di Monserrato, 09042 Monserrato, Cagliari, Italy; angela.corona@unica.it (A.C.); vonnis@unica.it (V.O.); francescaesposito@unica.it (F.E.); 2 Department of Molecular Medicine, University of Padova, 35121 Padova, Italy; claudia.delvecchio@unipd.it; 3 Department of Pharmacology, Yale University Medical School, New Haven, CT 06520-8066, USA; yccheng@yale.edu; 4 Genetics and Biomedical Research Institute, National Research Council, 09042 Monserrato, Italy

**Keywords:** HIV-1 therapeutic agents, RT dual inhibitors, HIV-1 ribonuclease H, nicotinic acid esters, nicotinic acid amide

## Abstract

The persistence of the AIDS epidemic, and the life-long treatment required, indicate the constant need of novel HIV-1 inhibitors. In this scenario the HIV-1 Reverse Transcriptase (RT)-associated ribonuclease H (RNase H) function is a promising drug target. Here we report a series of compounds, developed on the 2-amino-6-(trifluoromethyl)nicotinic acid scaffold, studied as promising RNase H dual inhibitors. Among the 44 tested compounds, 34 inhibited HIV-1 RT-associated RNase H function in the low micromolar range, and seven of them showed also to inhibit viral replication in cell-based assays with a selectivity index up to 10. The most promising compound, **21**, inhibited RNase H function with an IC_50_ of 14 µM and HIV-1 replication in cell-based assays with a selectivity index greater than 10. Mode of action studies revealed that compound **21** is an allosteric dual-site compound inhibiting both HIV-1 RT functions, blocking the polymerase function also in presence of mutations carried by circulating variants resistant to non-nucleoside inhibitors, and the RNase H function interacting with conserved regions within the RNase H domain. Proving compound **21** as a promising lead for the design of new allosteric RNase H inhibitors active against viral replication with not significant cytotoxic effects.

## 1. Introduction

The acquired immunodeficiency syndrome (AIDS) pandemic is still one of the major world health concerns. Presently approved treatments do not allow immunization of people or the eradication of the infection once acquired, and among the estimated 37.9 million people living with HIV worldwide in 2019, only 24.5 million people were accessing the current antiretroviral therapy [1]. While the highly-active antiretroviral therapy (HAART) allows patients who receive optimum treatment a life-expectation comparable to that of the uninfected population [2,3], the sub-optimal adherence and coverage of seropositive people, coupled with life-long treatment, still cause emergence of drug-resistant variants, that could lead to therapy failure [4], and whose transmission to naïve patient is a major concern and requires an optimized treatment [5,6,7]. These data remark the constant need to expand the antiretroviral armamentarium with new drugs, with novel targets, to be employed on salvage therapy. HIV-1 reverse transcriptase (RT) has been the first and most exploited target. RT is a heterodimeric (p66/p51) multifunctional enzyme that acts at the early stage of viral infection by converting the viral single-stranded RNA genome into a double-stranded DNA, with the concerted and dynamic interplay of its enzymatic functions RNA- and DNA-dependent DNA polymerase (RDDP and DDDP, respectively), and RT- associated ribonuclease H (RNase H) [8,9].

HIV-1 RT-associated RNase H is an enzymatic function present in all the members of the Ortervirales order [10] and it belongs to the nucleotidyltransferase family, characterized by a highly conserved Asp-Asp-Glu (DDE) motif, essential for coordination of two Mg^2+^ ions, and by a tertiary structure [11] conserved among its members [12,13,14,15]. HIV-1 RNase H function is essential for viral replication [16] and; therefore, is a good target for inhibitors [17]. Two decades of research identified many classes of compounds able to block its activity, that could be grouped in four big families according to their mode of action [17,18,19]: (i) Active site inhibitors, which chelate the two Mg^2+^ ions coordinated in the enzyme site by the DDE motif; (ii) RNase H selective allosteric inhibitors; (iii) allosteric inhibitors that bind at the p66/p51 interface; (iv) dual allosteric inhibitors of both the RDDP and RNase H functions. None of them has reached the clinical trial so far, enlightening the level of difficulty represented by the target [20]. 

The RNase H active-site inhibitors take advantage of the driving force represented by their chelating moiety to stably interact with the catalytic domain [21,22]; however, their development is often hampered by off-target toxicity and competition with nucleic acid substrates [23,24], and by the difficulties to establish strong additional interactions within the RNase H domain, although the possibility to target highly conserved residues in the surrounding molecular space has been successfully reported, with particular regard to the residues involved in the RNase H primer grip motif [25,26]. 

**Figure 1:**
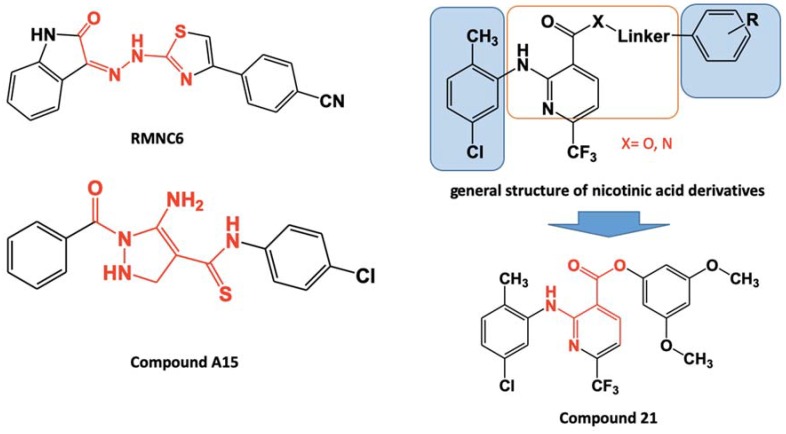
Structure of dual-site dual inhibitors. In red, the functionalized spacer; in black, the aromatic side portions. Summary of the 2-(arylamino)-6-(trifluoromethyl)nicotinic acid derivatives, in blue squares; the aromatic side portions, within red square.

The allosteric inhibition of the polymerase function of RT was achieved in the 1990s [27] with non-nucleoside inhibitors (NNRTI) that are still in the first-line therapy [28], although displaying a fairly low genetic barrier to selection of resistant variants, among which K103N, Y181C, and Y188L are the most common [29]. Differently, the first approaches to allosteric RNase H inhibition attempted to disrupt the enzyme stability by inhibiting the dimerization [30], and several efforts have been made to develop ligands able to bind at the interface between the heterodimer subunits [31,32,33], compromising RT heterodimer stability and flexibility, critical for its activity, as it is done by vinylogous ureas that bind in a site located in p51 subunit involved Cys280 and Lys281, located in α-helix H [34]. During the years also two other allosteric pockets have been identified within the p66 RT domain. The first one, firstly reported for the binding of the hydrazone DHBNH [35], it is located >50 Å away from the RNase H active site, between the RT polymerase active site and the polymerase primer grip, and partially overlaps with the NNRTI binding site. The second one, located between the RNase H active site and the region encompassing α-helices B and D in the RNase H domain, is centered on the residue Q507 [36,37]. Structure–activity relationship revealed that these two sites share common pharmacophoric requirements that have been summarized in two diverse substituted aromatic portions, bridged by a central core, either linear or made by heterocyclic rings, containing donors and acceptors of hydrogen bonds [38,39,40,41,42,43]. These features lead to the design of compound RMNC6 [42] (Figure 1) that has been proved to be a dual-site dual RT-functions inhibitor, able to block both RNase H and RDDP functions. More recently another compound, A15 (Figure 1), showed the same dual inhibitor profile, being also active against viral replication [40], hence embracing the promising polypharmacology approach to develop effective drugs less susceptible to the selection of resistant variants [44]. 

**Scheme 1:**
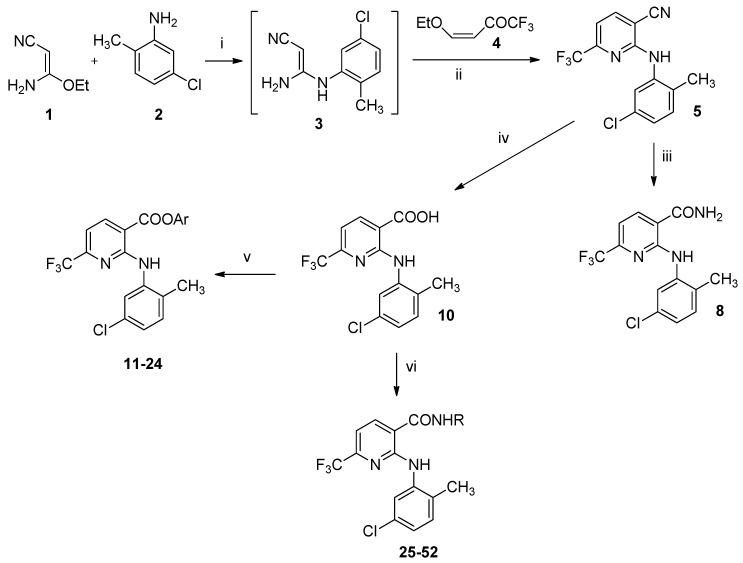
Synthetic routes to compounds **6**, **11**–**52**. (i) MeCN, r.t.; (ii) MeCN reflux; (iii) 20% aq NaOH, 100 °C; (iv) 50% aq H_2_SO_4_, 100 °C; (v) ArOH, EDCI, OHBt, MeCN, r.t.; vi) RNH_2_, EDCI, OHBt, MeCN, r.t.

Taking into account this information, we explored the 2-amino-6-trifluoromethyl-3-pyridinecarboxylic acid as a central core to design new allosteric RNase H inhibitors endowed with potential dual-target ability. Nicotinic and isonicotinic acid derivatives have been exploited as antimicrobial agents [45,46], although none of the molecules was tested on the RNase H function or was reported to be able to inhibit the viral replication. In the presented series of compounds, various aromatic portions are introduced as esters or amide of the nicotinic acid function, with an optional benzyl or piperazine linker (Scheme 1). Among the new 44 compounds, 34 inhibited HIV-1 RT-associated RNase H function in the low micromolar range, and seven of them showed also to inhibit viral replication in cell-based assays with a selectivity index up to 10, exhibited by compound **21** (Figure 1), the most promising derivative, that was investigated for its mode-of-action, displaying a dual-site, dual inhibition profile.

## 2. Results

### 2.1. Synthesis of 2-amino-6-(trifluoromethyl)nicotinate derivatives

**Scheme 2:**
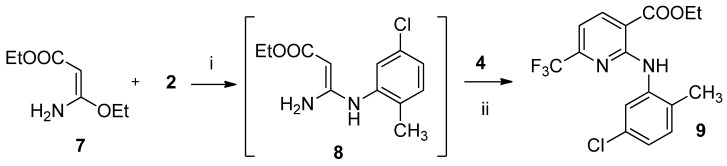
Synthetic route to compound **9**. (i) MeCN, r.t.; (ii) MeCN, reflux.

The target derivatives of 2-(2-methyl-5-chloro)-6-trifluoromethyl-3-pyridinecarboxylic acid **6**, **9**, **11**–**52** (Table 1) were synthesized as shown in Scheme 1 and Scheme 2. We have previously reported an easy and convenient method for the synthesis of 2-arylamino-6-trifluoromethyl-3-pyridinecarboxylic acids [47]. According to Scheme 1, 3-amino-3-ethoxypropenenitrile (**1**) was first treated with the 2-methyl-5-chloroaniline (**2**) in MeCN solution to give the non-isolable 3-amino-3-(2-methyl-5-chlorophenyl)aminopropenenitrile (**3**) and then with 1,1,1-trifluoro-4-iso-butoxy-3-buten-2-one (**4**). The temperature was gradually allowed to reach 60 °C. After 3 h, pyridinecarbonitrile **5** was obtained in good yields. Sodium hydroxide catalyzed hydrolysis of nitrile **5** afforded pyridine-3-carboxamide **6**. High yield of 6-trifluoromethyl-3-pyridinecarboxylic acid **10** was achieved by hydrolysis of derivative **5** in 50% aqueous sulfuric acid. Chemistry similar to that described in the synthesis of compound **5** was used for the preparation of ethyl ester **9 [48]**. As reported in Scheme 2, ethyl 3-amino-3-ethoxypropenoate (**7**) was sequentially treated with the amine **2**, then with enol ether **4** in MeCN solution to give the ester **9**. Treatment of acid **10** with the appropriate phenol in the presence of 1-(3-dimethylaminopropyl)-3-ethylcarbodiimide hydrochloride (EDCI) and hydroxybenzotriazole (HOBt) in MeCN solution gave esters **11**–**24** (Table 1) in 52$%–98% yields. Amide derivatives **25**–**52** (Table 1) were obtained in 39%–96% yields by the reaction of acid **10** with the appropriate amine by EDCI method [48]. All the newly synthesized compounds gave corrected analytical data. The IR and NMR spectral data were consistent with the assigned structure.

### 2.2. Evaluation of RNase H Inhibitory Activity

To establish the potential biologic effect of the synthesized compounds we tested them on the HIV-1 RT-associated RNase H activity, using as a control the RNase H inhibitors RDS1759 [25] and beta-Thujaplicinol (BTP) [49] (Table 1). The ethyl nicotinate **9** showed promising inhibitory activity (IC_50_ 24 μM) of the RNase H function. The replacement of ethoxy group with 4-substituted aryl rings, produced changes in potency, with the 4-chlorine (**13**), 4-methoxy (**18**) and 4-thiomethyl (**18**) aryl esters being the most potent of the ester series. Interestingly the shift of the 4-chlorine atom into 2- or 3-position led to drop in activity (esters **11** and **12**). The shift of the 4-hydroxy group of ester **18** into 2-position to give the analog **16** produced a small increase in inhibition potency, while the 3-hydroxyaryl ester **17** was less potent as compared to compounds **16** and **18**. The methylation of the hydroxy group of compound **18,** as well as its replacement with a thiomethyl group, (esters **23** and **24**, respectively), produced about two-fold enhancement of potency. On the contrary, the introduction of further methoxy groups, as in compounds **20**–**22**, slightly reduced the potency of inhibition. The introduction of a 2-chlorine atom on compound **13** to give the ester **14** abolished the activity, while the 2,4,6-trichlorophenyl ester **15** showed about four-fold reduction in potency as compared to ester **13**. The 3,5-dimethoxyphenyl ester **21** resulted about 1.5-fold more potent on the RNase H function as compared to the 3,4-dimethoxyphenyl analog **20**.

The amides showed a RNase H inhibitory activity rather different from esters. The nicotinamide **6** was completely inactive, while the oxime **25** showed more potent anti-RNase H activity, being the best inhibitor with an IC_50_ value of 0.7 μM. The monosubstituted aryl amides **28**–**30** and **37** inhibited RNase H activity with IC_50_ in the 5.6-20 μM range. The replacement of the 3-methyl group of amide **29** with a 3-methoxy group to give compound **33** abolished the activity, while the presence of two or three methoxy groups as in compounds **34**–**36** repristinated the anti-RNase H efficacy. The benzylamides **38**–**47** showed RNase H inhibitory activity (IC_50_ in the 9–24 μM concentration range). The arylpiperazino amides **49** and **51** displayed some RNase H inhibitory potency (IC_50_ values = 18 and 20 μM, respectively). The introduction of a second chlorine atom on amide **49**, to give the 3,4-dichlorophenyl piperazine analog **52**, produced an increase in potency (IC_50_ 7 μM).

### 2.3. Evaluation of Antiviral Activity 

The 28 compounds that were able to inhibit the HIV-1 RNase H activity with an IC_50_ value below 30 μM were tested for the ability to inhibit HIV-1 replication in TZMbl cells (Table 2). The 4-chlorophenyl ester **13** showed to be active on the HIV replication in the same range of concentration of the IC_50_ value, with an EC_50_ value of 10 μM, with a selectivity index (SI) of 3.6. The 3,4-dimethoxyphenyl ester **21** resulted as the best ester inhibitor of the HIV replication activity with an EC_50_ value of 5 μM and a SI greater than 10. Amides **34** and **36** showed to be able to inhibit HIV-1 replication (EC_50_ values = 2 and 1.8 μM, respectively) although residual cytotoxic effect activity caused a poor selectivity (SI = 2.6 and 1.8, respectively), and also the amides **49** and **52** displayed HIV inhibitory activity (EC_50_ values = 10 μM and SI = 2.7 and 1.6, respectively). Noteworthy, the most active RNase H inhibitor in biochemical assays, compound **25**, was inactive in the HIV replication assay.

### 2.4. Mode of Action Studies

**Figure 2:**
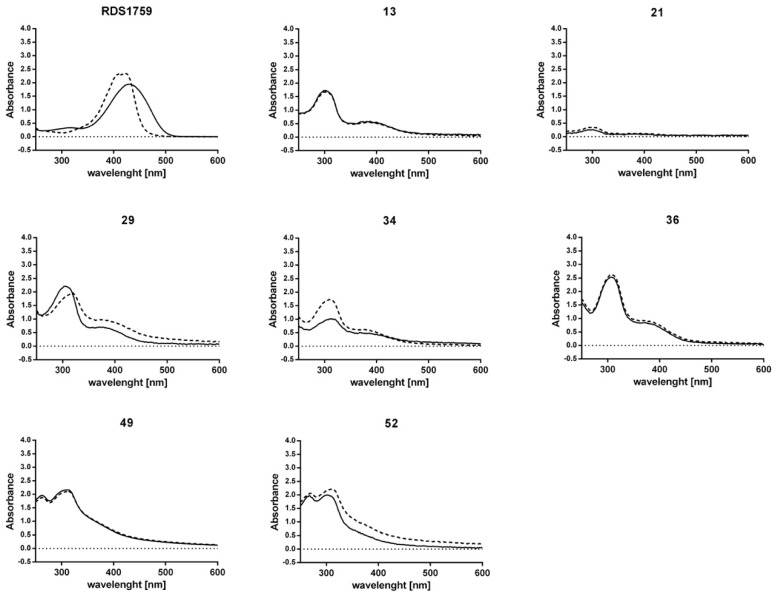
Effect of MgCl_2_ on the absorbance spectra of 2-(arylamino)-6-(trifluoromethyl)nicotinic acid derivatives. Mg^2+^ chelation UV-Vis spectrum was measured with 100 µM of compound alone (unbroken line) or in the presence of 6 mM MgCl_2_ (dotted line). The active site RNase H inhibitor RDS1759 was used as reference.

In order to confirm our hypothesis of allosteric binding mode, we firstly examined the possible interaction between selected compounds and the magnesium, used as a cofactor by both the RT-associated enzymatic functions. The UV spectra of the seven compounds able to inhibit viral replication were measured in the absence and in the presence of magnesium ions, using as a positive control the active site inhibitor **RDS1759**. Results (Figure 2) showed that, while the positive control displayed a typical shift in the maximum of absorbance of the spectra, among the tested compounds only amide **29** showed a shift of 17 nm in the peak of absorbance, while amides **34** and **52** showed a moderate hyperchromic effect, but not a shift. Differently, the spectra of compounds **13**, **21**, **36**, and **49** were not affected by the presence of the Mg^2+^. Overall, the data seem to exclude that Mg^2+^ coordination might be involved in the mechanism of action of the selected compounds.

In order to further dissect the mode of action the most promising compound, we selected ester **21** that inhibited the RNase H function with an IC_50_ value of 14 µM, the HIV-1 replication in cell culture with EC_50_ value of 5 µM and showed a SI ≥ 10, along with the amide derivative **49**, that was also active against HIV-1 replication but showed a higher toxicity in TZMbl cells. Both compounds were tested against the RDDP function (Table 3), showing IC_50_ values (16.1 and 22.9 µM, respectively), very similar to the ones obtained on the RNase H function, indicating a dual-function allosteric inhibitory profile. 

To clarify their binding mode, and also ascertain the possibility that these compounds could be active against circulating drug-resistant variants, compounds **21** and **49** were tested against both RT-associated functions of a panel of enzymes carrying single amino acid substitutions in the polymerase domain: mutations K103N, Y181C that are related to resistance to the NNRTIs [29], and mutation V108A that is related to the binding of the allosteric RNase H inhibitors on the pocket close to the NNRTI binding site [42]. Besides, both compounds were tested also on RTs mutated in two amino acid residues within the RNase H domain, Q475A and A502F, part of conserved regions of RNase H domain [50], that have been shown to be involved in the binding of RNase H inhibitors into a pocket close to the RNase H catalytic site but acting as allosteric RNase H inhibitors [25,37,40].

We tested the inhibition of the HIV-1 RDDP activity of mutated HIV-1 RTs by compounds **21** and **49** (Table 3), using as control the NNRTI efavirenz (EFV). Interestingly, the two compounds displayed a profile very different from EFV. Indeed, compounds **21** and **49** were able to inhibit the K103N and Y181C RT-associated RDDP activities. In particular, compound **21** showed no difference in potency of inhibition towards the two enzymes, while compound **49** showed a 2.5-fold decrease in potency of inhibition for K103N RT and a decrease of two-fold against Y181C RT. As expected, the IC_50_ value of EFV showed an increase of 7.6-fold against K103N, while it did not show change in potency of inhibition when tested against the V108A RT RDDP activity. Differently, compounds **21** and **49** showed a consistent increase in IC_50_ value (5.1- and >4.3-fold for compound **21** and **49**, respectively) on the V108A RT RDDP function. Of note, all the inhibitors were able to inhibit the RT-associated RDDP activity of A502F and Q475A RTs, except for **49** that showed to increase its RDDP IC_50_ value on Q475A RT of 2.5-fold.

The HIV-1 RNase H activity of the mutated HIV-1 RTs in the presence of compounds **21** and **49** was analyzed using as control the RNase H active site inhibitor **BTP** (Table 4). Results showed that the positive control equally inhibited all the tested enzymes, while compound **21** had a 3.7-fold increase in IC_50_ value on the K103N RT RNase H function, and it completely lost its RNase H inhibitory efficacy towards V108A, Q475A, and A502F RTs. Differently, compound **49** showed a moderate decrease in its potency of inhibition on the RNase H activity of the K103N, Y181C, and A502F RTs, being completely ineffective against the RNase H function of V108A and Q475A RTs.

Overall, results obtained on the V108A RT, whose RDDP and RNase H functions were both non-susceptible to inhibition by compounds **21** and **49,** indicate a major role played by the pocket close to the NNRTI binding site on inhibition of these compounds of both the enzymatic activities, with a combination of short- and long-range effects, and suggest a key role of residue V108 for the correct interaction between the inhibitors and the binding pocket. On the second hand, the fact that amino acidic substitutions Q475A and A502F caused a loss of potency only for inhibition of their RNase H functions suggest that this second allosteric pocket exerts a short-range influence only within the RNase H domain.

## 3. Discussion

Dual inhibitors are a new class of molecules that aim to increase the efficacy of the drug and raise the genetic barrier to overcome the selection of resistant strains by hitting two targets at the same time [44,51,52]. In the constant need to identify and develop new anti-HIV drugs, possibly active against circulating drug-resistant variants, this approach has been applied to RDDP/Integrase and RNase H/Integrase dual inhibitors [53,54,55,56,57,58,59,60,61], that target two different viral enzymes, and to RNase H/RDDP dual inhibitors, that target two distinct, but strongly interconnected, enzymatic functions located in the same enzyme [9,36,62].

In this work, we designed a set of 44 new compounds following the pharmacophoric requirements previously identified for RNase H/RDDP dual-site dual inhibitors [38,39,40,41,42,63]. In this case, the central core was represented by the 6-(trifluoromethyl)nicotinic acid, and, differently from the previous attempt to design nicotinate-based antimicrobial agents that had two aromatic functions [45,46], we decorated the core, making ester or amide of the nicotinic acid and introducing an arylamino moiety at the 2-position. The scaffold maintained a sp2 hybridization state of the linker [40,42], that in this case involved the aryl ring, with a steric hindrance slightly bigger than previously reported compounds. For the synthesis of the compounds, we successfully applied our previously reported method for the synthesis of 2-arylamino-6-trifluoromethyl-3-pyridine carboxylic acids [47], and the final esters and amides were obtained by EDCI method in good yields and high purity without use of a tedious purification procedure [48]. 

The designed molecules showed a consistent inhibition of the HIV-1 RT-associated RNase H function, with the amide derivatives being overall slightly more potent than the ester counterparts. Cell-based assays revealed that the scaffold is more promising than the previous reported attempts of RNase H/RDDP dual inhibitors. Among the 44 2-(arylamino)-6-(trifluoromethyl)nicotinic acid derivatives, 7 compounds inhibited viral replication, while no active compound has been identified so far among the isatin-derivatives [41,42]. Furthermore, among the pyrrole and pyrazole derivatives, only one dual inhibitor was active against viral replication [40], being less active (EC_50_ = 25 μM) and more toxic (CC_50_ = 44 μM). 

The 3,5-dimethoxyphenyl ester **21** was able to block HIV-1 replication with a SI > 10. The mode of action studies revealed that the compound acts differently form NNRTI inhibitors, with an activity profile against RDDP activity different from EFV on K103N and Y181C RTs, since these amino acid substitutions had a lower impact on its efficacy as compared to previously reported compounds [40,42,64]. Site-directed mutagenesis experiments showed that compound **21** binds independently to two different allosteric sites previously described: S first pocket (pocket 1), contiguous with the NNRTI binding pocket, was described for hydrazones [35], isatines [41,42] and pyrazoles [40]. A second pocket (pocket 2), within the RNase H domain, was firstly reported for selective allosteric RNase H inhibitors, such as hydrazones derivatives [36,37], and later for isatines [41,42] and pyrazoles [40]. For years the contribution of the two pockets to the inhibition of the two enzymatic functions was controversial. Our results confirmed that pocket 2 is an allosteric site for selective inhibition of HIV-1 RT-associated RNase H, since mutations A502F and Q475A completely abrogated the inhibitory effect of compound **21** on that sole function, proving also that these two conserved residues [50] play a key role on compound activity. Moreover, we showed that binding to pocket 1 is sufficient for inhibition of both enzymatic activities, with a combination of long- and short-range effects, as previously hypothesized [36,42], since the introduction of amino acidic substitution V108A had a strong impact on the inhibition of both RDDP and RNase H functions by compound **21**.

These results provide useful insights for the design of novel and more potent HIV-1 RT RNase H/RDDP allosteric dual inhibitors, active against selected drug-resistant variants. With this aim, a major focus should be pointed on pocket 1 as the most suitable to be exploited for drug design, and on compound **21** as a promising good hit to be developed.

## 4. Materials and Methods 

### 4.1. Chemistry

All commercially available solvents and reagents were used without further purification. NMR spectra were recorded on an Inova 500 spectrometer (Varian, Palo Alto, CA, USA). The chemical shifts (δ) are reported in parts per million downfield from tetramethylsilane (TMS), which was used as internal standard, and the spectra were recorded in hexadeuteriodimethylsulphoxide (DMSO-d_6_). Infrared spectra were recorded on a Vector 22 spectrometer (Bruker, Bremen, Germany) in Nujol mulls. The main bands are given in cm^−1^. Positive-ion electrospray ionization (ESI) mass spectra were recorded on a double-focusing MAT 95 instrument (Finnigan, Waltham, MA, USA) with BE geometry. Melting points (mp) were determined on a SMP1 Melting Point apparatus (Stuart Scientific, Stone, UK) and were uncorrected. All reported products showed NMR spectra in agreement with the assigned structures. The purity of the tested compounds was determined by combustion elemental analyses conducted by the Microanalytical Laboratory of the Department of Chemical and Pharmaceutical Sciences of the University of Ferrara with a MT-5 CHN recorder elemental analyzer (Yanagimoto, Kyoto, Japan), and the values found were within 0.4% of theoretical values. Compounds **6**, **9** [47] **11**–**15**, **19**, **21**, **23**, **27**, **29**–**37** and **49**–**51** were prepared as previously described [48].

#### 4.1.1. General Procedure for the Synthesis of Esters (**11**–**24**)

A mixture of acid **10** (0.33 g, 1 mmol), EDCI (1.92 g, 1.1 mmol), and HOBt (0.13 g, 1 mmol) in dry MeCN (10 mL) was stirred at room temperature for 30 min and then treated with the appropriate phenol (1 mmol). The mixture was stirred at room temperature for an additional 24 h. Then the solution was evaporated to dryness in vacuo. The residue was dissolved in ethyl acetate (20 mL) and washed with brine (2 × 5 mL), 5% aqueous sodium hydroxide (2 × 5 mL), and water (2 × 5 mL). The organic layer was dried over anhydrous sodium sulfate. Concentration of the dried extract yielded a residue which was triturated with di-isopropyl ether. The formed precipitate was filtered off and purified by crystallization from the adequate solvent to give the ester derivatives **11**–**24**.

*2-Hydroxyphenyl 2-((5-chloro-2-methylphenyl)amino)-6-(trifluoromethyl)nicotinate (**16**).* Yield 86%. Mp 179–180 °C (MeCN). ^1^H NMR (DMSO-d_6_): δ 2.38 (s, 3H, CH_3_), 6.98–7.41 (m, 5H, aryl), 7.52 d, 1H, *J* = 8.1 Hz, pyridyl), 7.46 (d, 1H, *J* = 8.4 Hz, aryl), 8.26 (d, 1H, *J* = 8.4 Hz, aryl), 8.88 (d, 1H, *J* = 8.1 Hz, pyridyl), 9.98 (s, 1H, NH), 10.06 (s, 1H, OH). IR (Nujol) 3330, 1712, 1615, 1590 cm^−1^. m/z 423 (M + H)^+^. Anal. Calcd for C_20_H_14_ClF_3_N_2_O_3_ (422.78) %C 56.82, %H 3.34, %N 6.63. Found %C 56.87, %H 3.32, %N 6.61.

*3-Hydroxyphenyl 2-((5-chloro-2-methylphenyl)amino)-6-(trifluoromethyl)nicotinate (**17**).* Yield 87%. Mp 188-190 °C (MeCN). ^1^H NMR (DMSO-d_6_): δ 2.27 (s, 3H, CH_3_), 7.30-7.66 (m, 7H, aryl), 8.11 (d, 1H, *J* = 8.5 Hz, aryl), 8.79 (d, 1H, *J* = 8.0 Hz, pyridyl), 9.88 (s, 1H, NH), 10.05 (s, 1H, OH). IR (Nujol) 3294, 1712, 1620, 1590 cm^−1^. m/z 423 (M + H)^+^. Anal. Calcd for C_20_H_14_ClF_3_N_2_O_3_ (422.78) %C 56.82, %H 3.34, %N 6.63. Found %C 56.78, %H 3.33, %N 6.60.

*4-Hydroxyphenyl 2-((5-chloro-2-methylphenyl)amino)-6-(trifluoromethyl)nicotinate (**18**).* Yield 63%. Mp 184-186 °C (EtOH). ^1^H NMR (DMSO-d_6_): δ 2.27 (s, 3H, CH_3_), 6.83 (d, 2H, *J* = 9.0 Hz, aryl), 7.13 (d, 2H, *J* = 7.0 Hz, aryl), 7.37 (m, 3H, aryl and pyridyl), 8.14 (d, 1H, *J* = 9.0 Hz, aryl), 8.73 (d, 1H, *J* = 7.5 Hz, pyridyl), 9.55 (s, 1H, NH), 9.86 (s, 1H, OH). IR (Nujol) 3474, 3345, 1687, 1617, 1589 cm^−1^. m/z 423 (M + H)^+^. Anal. Calcd for C_20_H_14_ClF_3_N_2_O_3_ (422.78) %C 56.82, %H 3.34, %N 6.63. Found %C 56.79, %H 3.36, %N 6.66.

*3,4-Dimethoxyphenyl-2-((5-chloro-2-methylphenyl)amino)-6-(trifluoromethyl)nicotinate (**20**).* Yield 52%. Mp 127-129 °C (2-propanol). ^1^H NMR (DMSO-d_6_): δ 2.30 (s, 3H, CH_3_), 3.79 (s, 3H, CH_3_), 3.86 (s, 3H, CH_3_), 6.85–7.98 (m, 6H, aryl and pyridyl), 8.24 (d, 1H, *J* = 8.5 Hz, aryl), 8.76 (d, 1H, *J* = 8.0 Hz, pyridyl), 10.51 (s, 1H, NH). IR (Nujol) 3345, 3278, 1748, 1699, 1622, 1608, 1591 cm^−1^. m/z 467 (M + H)^+^. Anal. Calcd for C_22_H_18_ClF_3_N_2_O_4_ (466.84) %C 56.60, %H 3.89, %N 6.00. Found %C 56.65, %H 3.90, %N 6.02.

*3,4,5-Trimethoxyphenyl 2-((5-chloro-2-methylphenyl)amino)-6-(trifluoromethyl)nicotinate (**22**).* Yield 98%. Mp 138-140 °C (2-propanol). ^1^H NMR (DMSO-d_6_): δ 2.26 (s, 3H, CH_3_), 3.67 (s, 3H, CH_3_), 3.76 (s, 6H, CH_3_), 6.73 (s, 2H, aryl), 7.28 (d, 1H, *J* = 8.5 Hz, aryl), 7.35–7.40 (m, 2H, aryl and pyridyl), 8.08 (d, 1H, *J* = 8.5 Hz, aryl), 8.72 (d, 1H, *J* = 7.5 Hz, pyridyl), 9.89 (s, 1H, NH). IR (Nujol) 3331, 3293, 1697, 1618, 1590 cm^−1^. m/z 497 (M + H)^+^. Anal. Calcd for C_23_H_20_ClF_3_N_2_O_4_ (496.86) %C 55.60, %H 4.06, %N 5.64. Found %C 55.54, %H 4.07, %N 5.66.

*4-(Methylthio)phenyl 2-((5-chloro-2-methylphenyl)amino)-6-(trifluoromethyl)nicotinate (**24**).* Yield 63%. Mp 125–127 °C (2-propanol). ^1^H NMR (DMSO-d_6_): δ (s, 3H, CH_3_), 7.41–7.52 (m, 10H, aryl and pyridyl), 8.24 (d, 1H, *J* = 8.8 Hz, aryl), 8.87 (d, 1H, *J* = 8.1 Hz, pyridyl), 10.03 (s, 1H, NH). IR (Nujol) 3294, 1712, 1620, 1590 cm^−1^. m/z 453 (M + H)^+^. Anal. Calcd for C_21_H_16_ClF_3_N_2_O_2_S (452.88) %C 55.69, %H 3.56, %N 6.19. Found %C 55.74, %H 3.58, %N 6.17.

#### 4.1.2. General Procedure for the Synthesis of Amides (**25**–**52**)

A mixture of acid **10** (0.33 g, 1 mmol), EDCI (1.92 g, 1.1 mmol), and HOBt (0.13 g, 1 mmol) in dry MeCN (10 mL) was stirred at room temperature for 30 min and then treated with the appropriate amine (1 mmol). The mixture was stirred at room temperature for an additional 24 h. Then the solution was evaporated to dryness in vacuo. The residue was dissolved in ethyl acetate (20 mL) and washed sequentially with brine (2 × 5 mL), 10% aqueous sodium carbonate (2 × 5 mL), 10% aqueous citric acid (2 × 5 mL), and water (2 × 5 mL). The organic layer was dried over anhydrous magnesium sulfate. Concentration of the dried extracts yielded a solid residue which was washed with ethyl ether, filtered off, and dried to give the amide derivatives in analytically pure form without additional purification by crystallization if not elsewhere indicated.

*2-((5-Chloro-2-methylphenyl)amino)-N-hydroxy-6-(trifluoromethyl)nicotinamide (**25**).* Yield 61%. Mp 154–155 °C. ^1^H NMR (DMSO-d_6_): δ 2.37 (s, 3H, CH_3_), 7.36–7.68 (m, 3H, aryl and pyridyl), 8.34 (d, 1H, *J* = 8.8 Hz, aryl), 8.86 (d, 1H, *J* = 7.7 Hz, pyridyl), 9.87 (s, 1H, NH), 10.59 (s, 1H, NH) 12.01 (s, 1H, OH). IR (Nujol) 3350, 1686, 1618, 1589 cm^−1^. m/z 346 (M + H)^+^. Anal. Calcd for C_14_H_11_ClF_3_N_3_O_2_ (345.70) %C 48.64, %H 3.21, %N 12.15. Found %C 48.69, %H 3.20, %N 12.18.

*Ethyl 2-(2-((5-chloro-2-methylphenyl)amino)-6-(trifluoromethyl)nicotinamido)acetate (**26**).* Yield 96%. Mp 128-130 °C. ^1^H NMR (DMSO-d_6_): δ 1.22 (t, *J* = 7.1 Hz, 3H, CH_3_), 2.39 (s, 3H, CH_3_), 4.27 (q, *J* = 7.1 Hz, 2H, CH_2_), 4.15 (d, *J* = 4.8 Hz, 2H, CH_2_), 7.25–7.39 (m, 3H, aryl and pyridyl), 8.20 (d, 1H, *J* = 8.0 Hz, aryl), 8.56 (d, 1H, J = 7.3 Hz, pyridyl), 9.59 (s, 1H, NH), 10.69 (s, 1H, NH). IR (Nujol) 3429, 1737, 1722, 1655 cm^−1^. m/z 416 (M + H)^+^. Anal. Calcd for C_18_H_17_ClF_3_N_3_O_3_ (415.79) %C 52.00, %H 4.12, %N 10.11. Found %C 52.05, %H 4.10, %N 10.08.

*2-((5-Chloro-2-methylphenyl)amino)-N-(4-(methylthio)phenyl)-6-(trifluoromethyl)nicotinamide (**28**).* Yield 64%. Mp 152-154 °C. ^1^H NMR (DMSO-d_6_): δ 2.27 (s, 3H, CH_3_), 2.48 (s, 3H, CH_3_), 7.23 (d, 1H, *J* = 8.5 Hz, aryl), 7.28 (m, 3H, aryl), 7.36 (d, 1H, *J* = 7.5 Hz, pyridyl), 7.65 (d, 2H, *J* = 7.5 Hz, aryl), 8.15 (d, 1H, *J* = 8.5 Hz, aryl), 8.46 (d, 1H, *J* = 7.5 Hz, pyridyl), 10.35 (s, 1H, NH), 10.64 (s, 1H, NH). IR (Nujol) 3380, 3300, 1639, 1588 cm^−1^. m/z 452 (M + H)^+^. Anal. Calcd for C_21_H_17_ClF_3_N_3_OS (451,89) %C 55.82, %H 3.79, %N 9.30.

*N-(2-chlorobenzyl)-2-((5-chloro-2-methylphenyl)amino)-6-(trifluoromethyl)nicotinamide (**38**).* Yield 93%. Mp 169-170 °C (2-propanol). ^1^H NMR (DMSO-d_6_): δ 2.39 (s, 3H, CH_3_), 4.72 (d, *J* = 5.2 Hz, 2H, CH_2_), 7.36–7.61 (m, 6H, aryl and pyridyl), 8.35 (d, 1H, *J* = 8.8 Hz, aryl), 8.56 (d, 1H, *J* = 8.0 Hz, pyridyl), 9.68 (s, 1H, NH), 10.90 (s, 1H, NH). IR (Nujol) 3300, 1636, 1613, 1597 cm^−1^. m/z 450 (M + H)^+^. Anal. Calcd for C_21_H_16_ClF_3_N_3_O (449.85) %C 58.74, %H 4.26, %N 9.34. Found %C 58.69, %H 4.27, %N 9.30.

*2-((5-Chloro-2-methylphenyl)amino)-N-(2-methoxybenzyl)-6-(trifluoromethyl)nicotinamide (**39**).* Yield 53%. Mp 148-150 °C (2-propanol). ^1^H NMR (DMSO-d_6_): δ 2.40 (s, 3H, CH_3_), 3.96 (s, 3H, CH_3_), 4.63 (d, *J* = 5.1 Hz, 2H, CH2), 7.02–7.48 (m, 6H, aryl and pyridyl), 8.37 (d, 1H, *J* = 8.6 Hz, aryl), 8.55 (d, 1H, *J* = 7.7 Hz, pyridyl), 9.52 (s, 1H, NH), 11.00 (s, 1H, NH). IR (Nujol) 3315, 1639, 1617, 1599 cm^−1^. m/z 450 (M + H)^+^. Anal. Calcd for C_21_H_16_ClF_3_N_3_O (449.85) %C 58.74, %H 4.26, %N 9.34. Found %C 58.79, %H 4.24, %N 9.37.

*N-(4-chlorobenzyl)-2-((5-chloro-2-methylphenyl)amino)-6-(trifluoromethyl)nicotinamide (**40**).* Yield 46%. Mp 130–131 °C (2-propanol). ^1^H NMR (DMSO-d_6_): δ 2.28 (s, 3H, CH_3_), 4.52 (d, *J* = 5.5 Hz, 2H, CH_2_), 7.24–7.40 (m, 7H, aryl and pyridyl), 8.23 (d, 1H, *J* = 9.0 Hz, aryl), 8.40 (d, 1H, *J* = 7.5 Hz, pyridyl), 9.60 (t, *J* = 5.5 Hz, 1H, NH), 10.96 (s, 1H, NH). IR (Nujol) 3283, 1634, 1612, 1599 cm^−1^. m/z 452 (M + H)^+^. Anal. Calcd for C_21_H_16_ClF_3_N_3_O (451.89) %C 55.52, %H 3.55, % N 9.25. Found %C 55.58, %H 3.54, % N 9.22.

*2-((5-Chloro-2-methylphenyl)amino)-N-(4-fluorobenzyl)-6-(trifluoromethyl)nicotinamide (**41**).* Yield 43%. Mp 184–185 °C (2-propanol). ^1^H NMR (DMSO-d_6_): δ 2.40 (s, 3H, CH_3_), 4.63 (d, *J* = 5.5 Hz, 2H, CH_2_), 7.25–7.54 (m, 7H, aryl and pyridyl), 8.35 (d, 1H, *J* = 8.7 Hz, aryl), 8.51 (d, 1H, *J* = 7.5 Hz, pyridyl), 9.69 (s, 1H, NH), 10.97 (s, 1H, NH). IR (Nujol) 3276, 1635, 1613 cm^−1^. m/z 438 (M + H)^+^. Anal. Calcd for C_21_H_16_ClF_4_N_3_O (437.82) %C 55.52, %H 3.55, %N 9.25. Found %C 55.57, %H 3.54, %N 9.23.

*2-((5-Chloro-2-methylphenyl)amino)-6-(trifluoromethyl)-N-(4-(trifluoromethyl)benzyl)nicotinamide (**42**).* Yield 62%. Mp 170–171 °C (2-propanol). ^1^H NMR (DMSO-d_6_): δ 2.38 (s, 3H, CH_3_), 4.74 (d, *J* = 5.5 Hz, 2H, CH_2_), 7.37 (d, 1H, *J* = 8.8 Hz, aryl), 7.43 (s, 1H aryl), 7.84 (d, 1H, *J* = 7.8 Hz, pyridyl), 7.70 (d, 2H, *J* = 8.3 Hz, aryl), 7.82 (d, 2H, *J* = 8.3 Hz, aryl), 8.34 (d, 1H, *J* = 8.8 Hz, aryl), 8.54 (d, 1H, *J* = 7.8 Hz, pyridyl), 9.78 (s, 1H, NH), 10.92 (s, 1H, NH). IR (Nujol) 3325, 1635, 1613, 1597 cm^−1^. m/z 488 (M + H)^+^. Anal. Calcd for C_21_H_16_ClF_4_N_3_O (487.83) %C 54.17, %H 3.31, %N 8.61.

*2-((5-Chloro-2-methylphenyl)amino)-N-(4-methoxybenzyl)-6-(trifluoromethyl)nicotinamide (**43**).* Yield 39%. Mp 126-128 °C (2-propanol). ^1^H NMR (DMSO-d_6_): δ 2.40 (s, 3H, CH_3_), 3.84 (2, 3H, CH_3_), 4.58 (d, *J* = 5.3 Hz, 2H, CH_2_), 7.02 (d, 1H, *J* = 7.1 Hz, aryl), 7.43 (s, 1H aryl), 7.34–7.45 (m, 6H, aryl and pyridyl), 8.35 (d, 1H, *J* = 8.8 Hz, aryl), 8.48 (d, 1H, *J* = 7.8 Hz, pyridyl), 9.63 (s, 1H, NH), 11.00 (s, 1H, NH). IR (Nujol) 3292, 1634, 1612, 1598 cm^−1^. m/z 450 (M + H)^+^. Anal. Calcd for C_22_H_19_ClF_3_N_3_O_2_ (449.85) %C 58.74, %H 4.86, %N 9.34. Found %C 58.68, %H 4.88, %N 9.37.

*2-((5-Chloro-2-methylphenyl)amino)-N-(4-methylbenzyl)-6-(trifluoromethyl)nicotinamide (**44**).* Yield 95%. Mp 189–190 °C (2-propanol). ^1^H NMR (DMSO-d_6_): δ 2.39 (s, 3H, CH_3_), 2.43 (s, 3H, CH_3_), 4.60 (d, *J* = 5.3 Hz, 2H, CH2), 7.25–7.47 (m, 6H, aryl and pyridyl), 9.66 (s, 1H, NH), 10.99 (s, 1H, NH). IR (Nujol) 3277, 1634, 1612, 1598 cm^−1^. m/z 434 (M + H)^+^. Anal. Calcd for C_22_H_19_ClF_3_N_3_O (433.85) %C 60.90, %H 4.41, %N 9.69. Found %C 60.97, %H 4.43, %N 9.66.

*2-((5-Chloro-2-methylphenyl)amino)-N-(2,4-dichlorobenzyl)-6-(trifluoromethyl)nicotinamide (**45**).* Yield 65%. Mp 153–155 °C (2-propanol). ^1^H NMR (DMSO-d_6_): δ 2.38 (s, 3H, CH_3_), 4.69 (d, *J* = 5.5 Hz, 2H, CH_2_), 7.36–7.57 (m, 5H, aryl and pyridyl), 7.76, (s, 1H, aryl), 8.34 (d, 1H, *J* = 8.7 Hz, aryl), 8.54 (d, 1H, *J* = 7.8 Hz, pyridyl), 9.69 (s, 1H, NH), 10.88 (s, 1H, NH). IR (Nujol) 3304, 1635, 1614, 1598 cm^−1^. m/z 489 (M + H)^+^. Anal. Calcd for C_21_H_15_ClF_3_N_3_O (488.72) %C 51.91, %H 3.09, %N 8.60. Found %C 51.96, %H 3.09, %N 8.64.

*2-((5-Chloro-2-methylphenyl)amino)-N-(3,4-dichlorobenzyl)-6-(trifluoromethyl)nicotinamide (**46**).* Yield 79%. Mp 179–180 °C (2-propanol). ^1^H NMR (DMSO-d_6_): δ 2.39 (s, 3H, CH_3_), 4.65 (d, *J* = 5.5 Hz, 2H, CH_2_), 7.27–7.82 (m, 6H, aryl and pyridyl), 8.34 (d, 1H, *J* = 8.8 Hz, aryl), 8.53 (d, 1H, *J* = 7.3 Hz, pyridyl), 9.80 (s, 1H, NH), 10.92 (s, 1H, NH). IR (Nujol) 3283, 1644, 1615, 1597 cm^−1^. m/z 489 (M + H)^+^. Anal. Calcd for C_21_H_15_ClF_3_N_3_O (488.72) %C 51.91, %H 3.09, %N 8.60. Found %C 51.85, %H 3.10, %N 8.63.

*2-((5-Chloro-2-methylphenyl)amino)-N-(4-hydroxy-3-methoxybenzyl)-6-(trifluoromethyl)nicotinamide (**47**).* Yield 95%. Mp 142–143 °C (2-propanol). ^1^H NMR (DMSO-d_6_): δ 2.33 (s, 3H, CH_3_), 3.85 (s, 3H, CH_3_), 4.53 (d, *J* = 5.4 Hz, 2H, CH_2_), 6.83–7.50 (m, 6H, aryl and pyridyl), 8.34 (d, 1H, *J* = 8.8 Hz, aryl), 8.54 (d, 1H, *J* = 7.8 Hz, pyridyl), 9.04 (s, 1H, OH), 9.60 (s, 1H, NH), 11.01 (s, 1H, NH). IR (Nujol) 3305, 1645, 1616 cm^−1^. m/z 466 (M + H)^+^. Anal. Calcd for C_22_H_19_Cl_3_F3N_3_O (465.85) %C 56.72, %H 4.11, %N 9.02. Found %C 56.67, %H 4.12, %N 9.06.

*(2-((5-Chloro-2-methylphenyl)amino)-6-(trifluoromethyl)pyridin-3-yl)(4-phenylpiperazin-1-yl)methanone (**48**).* Yield 81%. Mp 118–120 °C (n-hexane). ^1^H NMR (DMSO-d_6_): δ 2.25 (s, 3H, CH_3_), 3.17–3.21 (m, 4H, CH_2_), 3.36–3.41 (m, 4H, CH_2_), 6.80–7.32 (m, 8H, aryl and pyridyl), 7.55 (d, 1H, *J* = 8.5 Hz, aryl), 7.84 (d, 1H, *J* = 7.0 Hz, pyridyl), 8.55 (s, 1H, NH). IR (Nujol) 3365, 1630, 1614, 1599 cm^−1^. m/z 475 (M + H)^+^. Anal. Calcd for C_24_H_22_ClF_3_N_4_O (474.91) %C 60.70, %H 4.67, %N 11.80. Found %C 60.64, %H 4.69, %N 11.84.

*(2-((5-Chloro-2-methylphenyl)amino)-6-(trifluoromethyl)pyridin-3-yl)(4-(3,4-dichlorophenyl)piperazin-1-yl)methanone (**52**).* Yield 87%. Mp 165–167°C (MeCN). ^1^H NMR (DMSO-d_6_): δ 2.27 (s, 3H, CH_3_), 3.41–3.45 (m, 4H, CH_2_), 3.73–3.75 (m, 4H, CH_2_), 7.05–7.55 (m, 6H, aryl and pyridyl), 7.65 (d, 1H, *J* = 8.4 Hz, aryl), 7.96 (d, 1H, *J* = 7.7 Hz, pyridyl), 8.65 (s, 1H, NH). IR (Nujol) 3323, 1638, 1609 cm^−1^. m/z 544 (M + H)^+^. Anal. Calcd for C_24_H_20_Cl_3_F_3_N_4_O (543.80) %C 53.01, %H 3.71, %N 10.30. Found %C 53.07, %H 3.70, %N 10.27.

### 4.2. Biology

#### 4.2.1. Expression and Purification of Recombinant HIV-1 RTs Group M Subtype B

p6HRT-prot vector was kindly provided by Dr. Stuart Le Grice Laboratory. Heterodimeric RT was expressed essentially as described [42]. Briefly, *Escherichia coli* strain M15 containing the p6HRT-prot vector were grown up to an OD600 of 0.7 and induced with isopropyl β-D-1-thiogalactopyranoside (IPTG) 1.7 mM for 4 h. Cell pellets were resuspended in Lysis buffer (50 mM sodium phosphate pH 7.8, 0.5 mg/mL lysozyme), incubated on ice for 20 min, added 0.3 M final NaCl, sonicated and centrifuged at 30,000× *g* for 1 h. The supernatant was loaded into a Ni^2+-^sepharose column pre-equilibrated with loading buffer (50 mM sodium phosphate pH 7.8, 0.3 M NaCl, 10% glycerol, 10 mM imidazole) and washed thoroughly with wash buffer (50 mM sodium phosphate pH 6.0, 0.3 M NaCl, 10% glycerol, 80 mM imidazole). RT was gradient-eluted with elute buffer (wash buffer with 0.5 M imidazole). Fractions were collected, protein purity was checked by SDS-PAGE and found to be higher than 90%. RT containing fractions were pooled and diluted 1:1 with dilute buffer (50 mM sodium phosphate pH 7.0, 10% glycerol) then loaded into a Hi-trap Heparine HP GE (Healthcare Lifescience) pre-equilibrated with 10-column volume of loading buffer 2 (50 mM sodium phosphate pH 7.0, 10% glycerol, 150 mM NaCl). Column was then washed with loading buffer 2 and RT was gradient-eluted with elute buffer 2 (50 mM sodium phosphate pH 7.0, 10% glycerol, 150 mM NaCl). Fractions were collected, protein was dialyzed and stored in buffer containing 50 mM Tris HCl pH 7.0, 25 mM NaCl, 1 mM EDTA, 50% glycerol. Catalytic activities and protein concentration were determined. Enzyme-containing fractions were pooled and aliquots were stored at −80 °C.

#### 4.2.2. Mg^2+^ Coordination

The inhibitors were diluted in water to a final concentration of 100 μM. UV spectra recorded in the absence and after addition of 6 mM MgCl_2_ in a water solution, using a Nano Drop ONE reader (Thermo Scientific). Results are plotted as absorbance vs wavelength using Prisme 6, version 6.01.

#### 4.2.3. Site-Directed Mutagenesis

Amino acid substitutions were introduced into the p66 HIV-1 RT subunit coded in a p6HRT-prot plasmid using the QuikChange protocol (Agilent Technologies Inc., Santa Clara, CA).

#### 4.2.4. HIV-1 DNA Polymerase-Independent RNase H Activity Determination

The RT-associated RNase H activity of wt and mutated HIV RTs was measured as described [65], in 100 µL reaction volume containing 50 mM Tris HCl pH 7.8, 6 mM MgCl_2_, 1 mM dithiothreitol (DTT), 80 mM KCl, hybrid RNA/DNA (5’-GTTTTCTTTTCCCCCCTGAC-3’-Fluorescein, 5’-CAAAAGAAAAGGGGGGACUG-3’-Dabcyl), and different amounts of enzymes according to a linear range of dose-response curve—20 ng of wt RT; 50 ng K103N RT; 100 ng V108A RT; 4 ng Y181C RT; 100 ng Q475A RT; 100 ng A502F RT. The reaction mixture was incubated for 1 h at 37 °C, the reaction was stopped by addition of EDTA and products were measured with a Victor 3 (Perkin) at 490/528 nm.

#### 4.2.5. HIV-1 RNA-Dependent DNA Polymerase Activity Determination

The HIV-1 RT-associated RDDP activity was measured as described [66]. Briefly, in 25 µL volume containing 60 mM Tris-HCl pH 8.1, 8 mM MgCl_2_, 60 mM KCl, 13 mM DTT, poly(A)-oligo(dT), 100 µM dTTP, and 6 ng wt RT (or 21 ng K103N RT; 30 ng V108A RT; 1,5 ng Y181C RT; 21 ng Q475A RT; 15 ng A502F RT). After enzyme addition, the reaction mixture was incubated for 30 min at 37 °C and enzymatic reaction was stopped by addition of EDTA. Reaction products were detected by PicoGreen addition and measured with a Victor 3 (Perkin) at 502/523 nm.

#### 4.2.6. Antiviral Assay 

Drug-mediated inhibition of virus-induced cytotoxicity was assayed in TZM-BL cells. Triplicate wells of 96-well plates containing 1 × 10^4^ TZMbl cells were infected with HIV-1 IIIB strain at a multiplicity of infection of 0.1. Serial dilutions of drugs were added immediately after infection. Luciferase activity was measured as reported [67]. Two days after infection, the culture medium was removed from each well and 100 µl of Bright Glo reagent (Promega, Luis Obispo, CA) was added to the cells for measurement of luminescence using a Victor 2 luminometer (Perkin). The 50% effective concentration (EC_50_) was defined as the concentration that caused a 50% reduction of luciferase activity (relative light units) compared to virus control wells. Cell viability in CEM cells was quantified 5 days after infection with the MTT-dye reduction method.

## 5. Conclusions

We identified a new promising 2-amino-6-(trifluoromethyl)nicotinic acid scaffold for compounds that can inhibit both HIV-1 RT activities and are also active against viral replication. Compound **21**, active against viral replication in low micromolar range with no significant cytotoxic effects, showed to be a dual inhibitor active also against the RNA dependent DNA polymerase activity of HIV-1 RT, being active against RTs carrying NNRTI-resistant mutations. Mode-of-action and single-site mutagenesis studies indicated that compound **21** potency of inhibition is strongly impaired by the substitutions of amino acidic residues in two different pockets, located in two different domains of RT enzyme, hence supporting the hypothesis of a dual-site binding mode. Overall compound **21** is a promising lead for the design of new allosteric RNase H inhibitors more potent against HIV-1 replication.

## Figures and Tables

**Table 1:**
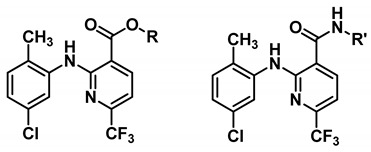
Biological effects of derivatives on HIV-1 Reverse Transcriptase (RT)-associated RNase H function.

Compound	R	R’	RNase H IC_50_ (µM) ^a^
**6**		H	>100 (87%) ^b^
**9**	OEt		24.0 ± 1.7
**11**	2-Cl-phenyl		>100 (92%)
**12**	3-Cl-phenyl		>100 (98%)
**13**	4-Cl-phenyl		9.3 ± 2.5
**14**	2,4-Cl_2_-phenyl		>100 (98%)
**15**	2,4,6-Cl_3_-phenyl		31.8 ± 3.5
**16**	2-OH-phenyl		16.2 ± 6.6
**17**	3-OH-phenyl		26.6 ± 5.6
**18**	4-OH-phenyl		20.0 ± 1.3
**19**	3-OCH_3_-phenyl		>100 (80%)
**20**	3,4-(OCH_3_)_2_-phenyl		21 ± 11
**21**	3,5-(OCH_3_)_2_-phenyl		14.7 ± 1.4
**22**	3,4,5-(OCH_3_)_3_-phenyl		17.3 ± 1.3
**23**	4-OCH_3_-phenyl		11.6 ± 4.3
**24**	4-SCH_3_-phenyl		9.6 ± 0.8
**25**		OH	0.77 ± 0.06
**26**		CH_2_COOEt	>100 (100%)
**27**		3-pyridyl	25.0 ± 4.9
**28**		4-SCH_3_-phenyl	5.9 ± 0.9
**29**		3-CH_3_-phenyl	8.9 ± 0.1
**30**		3-Cl-phenyl	4.0 ± 2.2
**31**		2,4-Cl_2_-phenyl	8.8 ± 0.5
**32**		3-Cl, 4-OCH_3_	>100 (100%)
**33**		3-OCH_3_-phenyl	>100 (54%)
**34**		3,5-(OCH_3_)_2_-phenyl	17.5 ± 3.9
**35**		3,4-(OCH_3_)_2_-phenyl	22.4 ± 4.0
**36**		3,4,5-(OCH_3_)_3_-phenyl	19.4 ± 1.5
**37**		4-CF_3_-phenyl	7.8 ± 1.2
**38**		2-Cl-benzyl	19.4 ± 1.1
**39**		2-OCH_3_-benzyl	18.7 ± 1.7
**40**		4-Cl-benzyl	6.4 ± 0.5
**41**		4-F-benzyl	23.3 ± 3.1
**42**		4-CF_3_-benzyl	21.9 ± 0.2
**43**		4-OCH_3_-benzyl	30.4 ± 3.9
**44**		4-CH_3_-benzyl	21.3 ± 0.5
**45**		2,4-Cl_2_-benzyl	19.3 ± 0.6
**46**		3,4-Cl_2_-benzyl	25.6 ± 3.4
**47**		3-OCH_3_, 4-OH-benzyl	18.2 ± 1.7
**48**		4-phenylpiperazino	>100 (100%)
**49**		4-(3-Cl-phenyl)piperazino	18.0 ± 5.7
**50**		4-(4-F-phenyl)piperazino	>100 (100%)
**51**		4-(4-OCH_3_-phenyl)piperazino	20.8 ± 2.5
**52**		4-(3,4-Cl_2_-phenyl)piperazino	7.5 ± 2.0
**RDS1759**	-	-	8.5 ± 0.5
**BTP**	-	-	0.19 ± 0.03

^a^ Compound concentration (± standard deviation) required to inhibit HIV-1 Reverse Transcriptase (RT)-associated RNase H activity in cells by 50%. ^b^ Numbers in parenthesis represent the percentage of cells viability in presence of the highest concentration of inhibitor tested.

**Table 2:** Biological effects of derivatives on HIV-1 replication.

Compoud	HIV-1 ^a^ EC_50_ (μM)	^b^ CC_50_ (μM)		SI ^d^
	TZMbl Cells	TZMbl	CEM Cells	
**9**	>10	>50 (75%) ^c^	>50 (100%)	-
**13**	10	36 ± 1	>50 (70%)	3.6
**16**	>10	12.2 ± 2.1	>12.5 (100%)	-
**17**	>50	>50 (62%)	>50 (100%)	-
**21**	5 ± 0.8	>50 (59%)	>50 (100%)	>10
**23**	>10	>50 (70%)	>50 (100%)	-
**24**	>10	50	>50 (100%)	-
**25**	>10	14 ± 2.8	35	-
**27**	>5.8	5.8 ± 0.8	16.2	-
**29**	2.5 ± 0.4	3.9 ± 0.9	6.3	1.6
**30**	>3.9	3.9 ± 0.9	8.4	-
**31**	>4.7	4.7 ± 1.7	7.8	-
**34**	2 ± 0.3	5.3 ± 0.6	10.4	2.6
**36**	1.8 ± 0.6	2.2 ± 0.4	10.2	1.2
**37**	>2.2	2.2 ± 0.2	4.3	-
**38**	>5.7	5.7 ± 0.7	12.5	-
**39**	>10	14.8 ± 3.1	>12.5 (55%)	-
**40**	>4.7	4.7 ± 0.4	7.75	-
**41**	>6	6 ± 0.6	11.6	-
**42**	>3.6	3.6 ± 1.2	5	-
**43**	>10	13.2 ± 1.3	20	-
**44**	>1.1	1.1 ± 0	3.5	-
**45**	>10	5 ± 0.6	9.15	-
**46**	>6.3	6.3 ± 1.3	10.6	-
**47**	>10	15 ± 3	17.4	-
**49**	10 ± 1.5	27 ± 3	>50 (82%)	2.7
**51**	>10	27.7 ± 12.8	>50 (81%)	-
**52**	10 ± 1.2	15.7 ± 5.1	>50 (76%)	1.6
**EFV**	0.15 ± 0.02	>50 (100%)	>50 (100%)	357

^a^ Effective concentration 50 (EC_50_): compound concentration required to decrease viral replication in cells by 50%. ^b^ Citotoxic concentration 50 (CC_50_): ompound concentration required to reduce infected cells viability by 50%. ^c^ Numbers in parenthesis represent the percentage of cells viability in presence of the highest concentration of inhibitor tested. ^d^ Selectivity index.

**Table 3:** Inhibition of HIV-1 RDDP activity of mutated HIV-1 RTs.

	21		49		EFV	
RT	IC_50_ (µM) ^a^	Fold^c^	IC_50_ (µM) ^a^	Fold	IC_50_ (nM) ^a^	Fold
WT	16.1 ± 1.2		22.9 ± 1.4		23 ± 4.1	1
K103N	16.5 ± 4.8	1.0	68.6 ± 3.0	2.5	176 ± 25	7.6
Y181C	22.5 ± 5.3	1.4	46.4 ± 2.4	2.0	49.7 ± 9.1	2.2
V108A	82.8 ± 6.3	5.1	>100 (54%) ^b^	> 4.3	21.3 ± 3.6	0.9
Q475A	17.0 ± 4.5	1.0	57.7 ± 2.1	2.5	34.5 ± 2.3	1.5
A502F	8.0 ± 0.7	0.5	27.0 ± 1.7	1.2	24.7 ± 2.4	1.1

^a^ Inhibitory Concentration 50 (IC_50_): compound concentration (± standard deviation) required to inhibit HIV-1 Reverse Transctiptase (RT)-associated RDDP activity in cells by 50%. ^b^ Numbers in parenthesis represent the percentage of cells viability in presence of the highest concentration of inhibitor tested. ^c^ Fold: ratio between the IC_50_ showed by the compound against the mutated RT and the IC_50_ against the wt RT.

**Table 4:** Inhibition of HIV-1 RNase H activity of mutated HIV-1 RTs.

	21		49		BTP	
RT	IC_50_ (µM) ^a^	Fold	IC_50_ (µM)	Fold	IC_50_ (µM)	Fold
WT	14.7 ± 1.4		18.0 ± 5.7		0.19 ± 0.03	1
K103N	53.9 ± 2.2	3.7	41.0 ± 2.2	2.3	0.22 ± 0.08	1.2
Y181C	11.9 ± 1.4	0.8	55.4 ± 12.6	3.1	0.23 ± 0.05	1.2
V108A	>100 (100%) ^b^	>6.8	>100 (100%)	>5.5	0.34 ± 0.4	1.8
Q475A	>100 (100%)	>6.8	>100 (100%)	>5.5	0.19 ± 0.03	1.0
A502F	>100 (55%)	>6.8	47.7 ± 5.4	2.6	0.17 ± 0.03	0.9

^a^ Compound concentration (± standard deviation) required to inhibit HIV-1 RT-associated RNase H activity in cells by 50%.^b^ Numbers in parenthesis represent the percentage of cells viability in presence of the highest concentration of inhibitor tested.
